# Evaporation, spreading, and possible uptake of droplets on sorghum (*Sorghum bicolor*) and cowpea (*Vigna unguiculata*) leaves using an imaging‐based technology

**DOI:** 10.1002/ps.8491

**Published:** 2024-10-29

**Authors:** Iaroslav Makhnenko, Cody Hoerning, Dustyn D Sawall, Steven A Fredericks, Elizabeth R Alonzi, Cari S Dutcher

**Affiliations:** ^1^ Department of Mechanical Engineering University of Minnesota Twin Cities Minneapolis MN USA; ^2^ Winfield United River Falls WI USA; ^3^ Department of Chemical Engineering and Materials Science University of Minnesota Twin Cities Minneapolis MN USA

**Keywords:** droplet, evaporation, spreading, contact angle, sorghum, cowpea

## Abstract

**BACKGROUND:**

Sprayed agrochemical droplets have a dynamic evolution on the leaf surface, undergoing changes in shape and volume due to spreading, evaporation, and adsorption. To better understand these processes, an accessible imaging‐based experimental methodology is presented to precisely measure droplet spreading, evaporation, and potential uptake by a leaf within a controlled relative humidity environment. Laboratory experiments were conducted to determine the effect of hydrocarbon surfactants, accelerators (light mineral oil), and humectants (high fructose corn syrup) on droplet spread, evaporation, and potential uptake when applied to sorghum (*Sorghum bicolor ‘Tricker’*) and cowpea *(Vigna unguiculata*) leaves.

**RESULTS:**

Experiments on cowpea leaves showed uniform spreading and no change in evaporation compared to the predicted rate. In contrast, on sorghum leaves, results suggest that the volume loss rate exceeds the predicted evaporation rate (up to 23%), indicating a potential uptake by the leaves. Some accelerated dynamics on sorghum can be attributed to the lateral spreading observed on hairy leaves along the veins, increasing the contact area by an average of 65%. However, samples containing light mineral oil, typically considered an accelerant to aid in uptake, demonstrated the highest rate but exhibited minimal spreading.

**CONCLUSIONS:**

The study demonstrates how droplet composition affects droplet dynamics on waxy and hairy leaves by using an imaging‐based methodology to measure evaporation rates, volume loss, contact angle, wetted area, and spreading behavior. The findings highlight some of the complex coupling between the crop protection product composition and droplet life cycle on a leaf. © 2024 The Author(s). *Pest Management Science* published by John Wiley & Sons Ltd on behalf of Society of Chemical Industry.

## Graphical abstract

Laboratory experiments were conducted to determine the effect of adjuvants on droplet spread, evaporation, and potential uptake on sorghum and cowpea leaves within a precisely controlled relative humidity environment.

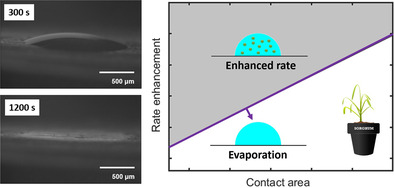

## 1 INTRODUCTION

Plants engage in a dynamic battle for survival. To combat external threats, plants have an array of defenses. Primary among them are plant defensive tissues. The epidermis and the cuticle serve as protective layers against the external environment.^1^, ^2^, ^3^, ^4^, ^5^ These barriers impede the deposition, retention, spreading, and penetration of crop protection products. One tool to enhance plant uptake of crop protection products is chemical adjuvants, defined as non‐active ingredients added to or already included in agrichemical formulation to improve performance. Adjuvants such as surfactants and oils added to improve spray breakup^6^, ^7^, ^8^, ^9^ and deposition behavior have been shown to be effective in some cases overcoming this barrier.^10^, ^11^, ^12^, ^13^, ^14^, ^15^, ^16^ The use of adjuvants containing humectants, such as glycerol, polysorbate, or polypropylene glycol, helps in preventing the rapid drying of pesticide droplets on leaf surfaces, further enhancing their overall effectiveness.^12^, ^17^, ^18^ However, it is important to note that the effectiveness of adjuvants can vary depending on the specific crop protection product, target plant, and environmental conditions.^19^, ^20^

Adjuvants often contain surfactants that significantly influence the surface tension of spray droplets at the air–liquid interface and the contact angle at the liquid–plant interface. Surfactants used in crop protection products can vary in charge, including nonionic, cationic, anionic, and zwitterionic. They also differ in type, encompassing hydrocarbon‐ and siloxane‐based structures. These surfactants lower the surface tension of spray droplets on leaf surfaces, and consequently increase droplet coverage and foliar uptake.^1^, ^14^, ^21^ The surfactant concentration can also influence the efficacy of agrochemical application. According to Wang and Liu,^1^ an increase in surfactant concentration from 0.01% to 1% (v/v) resulted in an enhanced overall foliar uptake of pesticides across a variety of studies.

The wetting dynamics of spray droplets also depend on the leaf surface.^22^ Leaves that are resistant to wetting commonly have waxy and hairy surfaces, resulting in an increased water‐repellent nature. Epicuticular waxes can take on crystalline, amorphous, or intermediate structures. Leaves that possess crystalline wax structures tend to be particularly hydrophobic, making them challenging to wet when sprayed with an aqueous solution.^23^, ^24^ Leaves with tiny hair‐like structures called trichomes also repel water more effectively than leaves without trichomes. Trichomes on leaf surfaces enhance water repellency by affecting the contact angle of droplets,^25^ and they may also prevent droplets from reaching the epidermis, resulting in relatively low droplet retention on leaves.^26^

Whether leaves have a waxy or hairy surface, the aqueous solution applied in the form of spray droplets must subsequently penetrate the leaf's hairs and waxy coatings. Afterward, it should penetrate the leaf tissue by crossing cell walls and/or entering through stomata.^27^ Consequently, the leaf's wettability plays a pivotal role in the processes of deposition, retention, and spread of spray droplets on the leaf surface, as well as in enabling the penetration of pesticides into the leaf.

Understanding the dynamic evolution of droplets on a leaf surface is crucial in agricultural research. When droplets remain on a leaf surface for a longer period of time, this enhances the adsorption and uptake of active ingredients.^28^ The objective of this research is to advance the field by developing and employing a novel experimental methodology for the precise measurement of droplet spreading and evaporation on a leaf surface, as well as potential uptake by a leaf, within a precisely controlled relative humidity environment. The present study considers the influence of droplet constituents, including combinations of a surfactant, a mineral oil, and high fructose corn syrup, on the dynamic behavior of droplets on both waxy and hairy leaves through the measurement of rates of evaporation and volume loss, contact angle, wetted area, and the spreading behavior of the droplets.

## 2 MATERIALS AND METHODS

### 2.1 Plant growing conditions

This study was conducted on leaf surfaces from two plant species: *Sorghum bicolor ‘Tricker’* (sorghum), a plant with hairy leaves, and *Vigna unguiculata* (cowpea), a plant with waxy leaves, that were grown in a greenhouse at the Winfield United Innovation Center in River Falls, WI (44°53′52.3″, −92°38′26.8″). Plants were sown at a depth of 3.8 cm in soil‐less potting mix (Carlin Horticultural Supplies, Milwaukee, WI, USA) with 1 g of slow‐release fertilizer (Osmocote Bloom, 13‐7‐18 NPK; Everris NA Inc, Dublin, OH, USA) in 11.4‐cm square pots and irrigated daily to water‐holding capacity. Greenhouse conditions were maintained at 28° C during the day (07:00 to 20:00) and 23 °C during the night. Relative humidity was maintained at 25–35% throughout the day. Supplemental lighting was provided during the daytime when the photosynthetically active radiation (PAR) level supplied by sunlight fell below 250 μmol PAR/m^2^s. Plants were grown for 21–25 days under these greenhouse conditions before being transferred to a laboratory setting for testing. The laboratory experiments were conducted within 30 days after transfer from the greenhouse (refer to the Supporting Information for details on the sorghum plants under different growth conditions). The analyses were conducted on the adaxial surface of the newest fully developed leaf.

### 2.2 Samples used

A total of three distinct adjuvant types, namely surfactant, humectant, and accelerator, were used in various combinations with deionized (DI) water. This work uses the water‐soluble surfactant Triton X‐100 (t‐octylphenoxypolyethoxyethanol), a nonionic surfactant, (CAS 9002‐93‐1; Sigma‐Aldrich, St. Louis, MO, USA), which has a density of 1.07 g/mL. The humectant consists of high fructose corn syrup (CAS 977042‐84‐4) with a density of 1.35 g/mL. The accelerator consists of light mineral oil (abbreviated as LMO, CAS 8042‐47‐5; Sigma‐Aldrich, St. Louis, MO, USA), which has a density of 0.84 g/mL.

The combinations of surfactant, humectant, and accelerator considered in the present study are summarized in Table 1. Each composition was tested 10 times, and the volume of each droplet tested was ~0.2 uL, which corresponds to ~0.73‐mm droplet diameter. Mixtures of these components were used to make droplets that were deposited on the surface of various substrates to measure the contact angle, rate of evaporation, rate of potential uptake by a leaf, and increase in contact area after spreading.

**Table 1:** Summary of four chemical compositions of the model adjuvant systems

Solution	Solution composition
1	Water
2	Water + 100 ppm v/v Triton X‐100
3	Water + 100 ppm v/v Triton X‐100 + 1% v/v LMO
4	Water + 100 ppm v/v Triton X‐100 + 1% v/v high fructose corn syrup

### 2.3 Experimental set‐up

**Figure 1:**
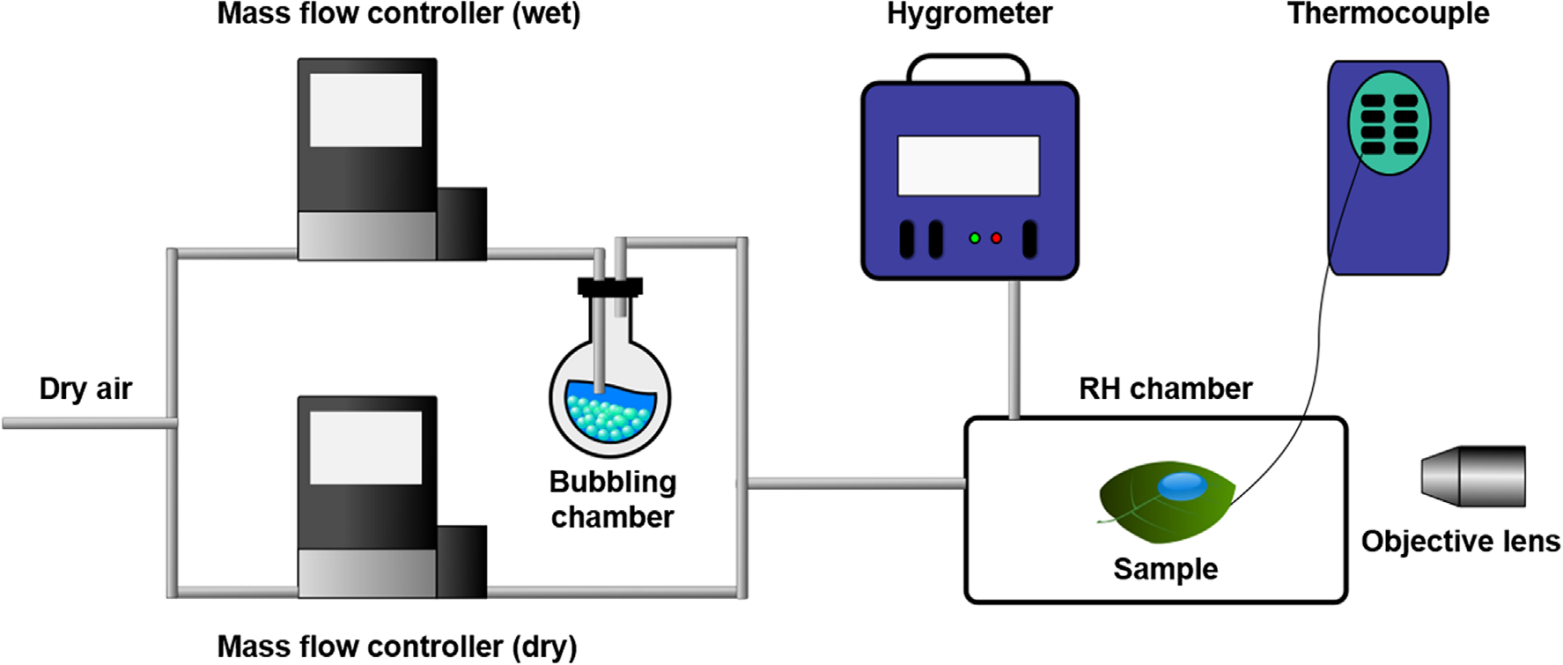
Schematic of the relative humidity (RH) stage.

To ensure consistent humidity, the experiments were carried out in a relative humidity (RH) chamber (Fig. 1). The humidity chamber was equipped with gas inlets and an exhaust line that was connected to a hygrometer (Buck, CR‐4) and a thermocouple (Pico, TC‐08), enabling precise measurement of RH in the chamber. Control of the RH was achieved by passing nitrogen gas (N_2_) through a sequence of glass beads containing DI water, while adjusting the proportion of wet to dry N_2_ entering the RH chamber^29^ using Alicat mass flow controllers. All experiments were conducted at an RH level of 78–82%.

To verify accurate humidity control, a series of humidity uptake measurements was performed with the well‐characterized inorganic model systems of NaCl and KCl. Prior to each experiment, the samples were dissolved in DI water and deposited onto a glass slide, which was then placed in the humidity chamber. Following this preparation, the RH was incrementally decreased from 95% to 30%, then increased from 30% to 95% using 2–3% step changes followed by 10‐minute equilibration periods. The change in RH was achieved by varying the ratio of wet to dry flow into the chamber, and the temperature and dew point data obtained from the hygrometer were utilized to calculate the RH within the chamber:

(1) $$\mathrm{RH}=100\%\frac{{\mathrm{e}}^{\left(18.678-\frac{\mathrm{DP}}{234.5}\right)\frac{\mathrm{DP}}{\mathrm{DP}+257.14}}}{{\mathrm{e}}^{\left(18.678-\frac{T}{234.5}\right)\frac{T}{T+257.14}}}$$

where DP and *T* are the dew point and temperature in Celcius, respectively.^30^, ^31^ For NaCl, efflorescence (or crystallization) occurred around 45–47% RH, while deliquescence (or humidification) occurred around 73–75% RH. For KCl, efflorescence occurred at 85%, and deliquescence occurred at 53%. These experimental values closely align with the reported literature values.^32^, ^33^

### 2.4 Droplet imaging and mathematical model

**Figure 2:**
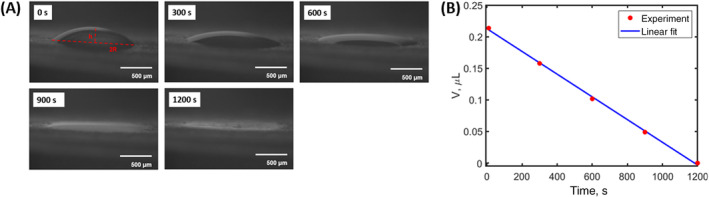
(A) Evaporation of a water droplet containing 100 ppm of surfactant and 1% of high fructose corn syrup at 80% RH. (B) Corresponding volume measurements as a function of time along with linear fit used to determine the volume loss rate of 0.179 nL/s.

For droplet imaging, leaves were detached from the plant and droplets were placed onto their surface with a syringe within minutes of the detachment. Samples on recently detached leaves yielded similar results to those on attached leaves (see Supporting Information, Fig. S4) and were significantly easier to work with when detached. The sample was placed inside the relative humidity chamber with a light source behind it and imaged from the side using a Basler camera (acA1300‐60 g) with a multi‐configurable macro lens. The volume of the droplet was measured by identifying the radius of the contact area and the height of the spherical cap through manual inspection using image processing software ImageJ^34^ (Fig. 2(A)) and using Eqn (3) at five points. The data were then fitted with a linear trendline using the least square method, with the slope of the line corresponding to the volumetric loss rate, which is a sum of the evaporation rate and a possible leaf uptake rate. (Fig. 2(B)). The *x*‐axis is the time after the sample was placed on the leaf surface and put in the humidity chamber.

To determine the appropriate shape to model the droplets, the capillary length was calculated via:

(2) $$k^{-1}=\sqrt{\gamma_{\mathrm{LG}}/\mathrm{\rho g}}$$

where, $\gamma_{\mathrm{LG}}$ is the surface tension of the liquid–gas interface, *ρ* is the fluid density, and *g* is the gravitational acceleration. At room temperature for water in air, $k^{-1}$≃3 mm, and for water with surfactant at critical micelle concentration, $k^{-1}$≃2 mm, which are both larger than the radius of the sessile droplets in our experiments (less than 1 mm), thereby allowing us to consider the shape of these droplets to be a spherical cap. By describing the droplet's shape as a spherical cap, it can be uniquely characterized using just two parameters: the radius (R) of the contact area and the height (h) of the spherical cap (see labels in Fig. 2(A)). Geometrically, these parameters are related to the droplet volume (V) through the equation:

(3) $$V=\frac{1}{6}\mathrm{\pi h}\left(3R^{2}+h^{2}\right).$$

In this scenario, the contact angle of the spherical cap can be expressed as:

(4) $$\theta =2\tan^{-1}\left(\frac{h}{R}\right).$$

We assume a quasi‐stationary isothermal vapor diffusion into the surrounding gas phase governs the evaporation process, with the assumption that the droplet is in contact with an infinite volume of gas. Thermal effects resulting from fluid evaporation are neglected, assuming that heat transfer occurs on significantly shorter timescales compared to those associated with the evaporation process. In the case of water in air, the evaporative cooling at the droplet's surface exerts a minimal influence on the evaporation rate. Consider the simplest case of a spherical single‐component liquid droplet suspended in the air, far removed from any surface, where the rate (magnitude) of volume change due to diffusion‐limited evaporation is described as follows:

(5) $$\frac{\mathrm{d}V}{\mathrm{d}t}=\frac{4\pi R_{s}\mathrm{D\Delta c}}{\rho }$$

where *V* is the volume, $R_{s}$ is the spherical radius of the droplet, *D* is the diffusion coefficient of water vapor in air, Δc is the difference in vapor mass concentration between the vicinity of the droplet and the distant region $\mathrm{\Delta c}=c_{0}-c_{\infty }$, and *ρ* is the density of the liquid.^35^, ^36^, ^37^ At the liquid–air interface, the vapor concentration $c_{0}$ is assumed to be equal to the saturation vapor concentration $c_{v}$. Far away from the droplet, the vapor concentration is equal to the ambient value $\mathrm{RH}c_{v}/100$, and the water vapor concentration gradient, $\mathrm{\Delta c}=c_{v}\left(1-\mathrm{RH},/,100\right)$, drives the evaporation.

A slight change in the evaporation model of a sphere is needed for sessile droplets. In addition to the change in the droplet geometry, the evaporation process affected by diffusion also experiences variations in the available space for vapor diffusion. Consequently, even a fully spherical droplet in contact with a flat solid surface evaporates at a different rate compared to a spherical droplet far away from the surface. Hu and Larson^38^ conducted a study on sessile droplet evaporation and presented a solution for the diffusion‐limited evaporation:

(6) $$\frac{\mathrm{d}V}{\mathrm{d}t}=\frac{\text{πRDΔc}\left(0.27\theta^{2}+1.3\right)}{\rho }$$

where R is the radius of the contact line and θ is the contact angle in radians. According to Hu and Larson,^38^ when the contact angle is below 40°, the angle does not have a significant effect on the rate, making the evaporation rate constant. For our analysis, the initial contact angle is used to estimate the predicted evaporation rate, which is calculated by identifying R and h (Fig. 2(A) at 0 s) and using Eqn (4).

### 2.5 Calibration of the model

**Figure 3:**
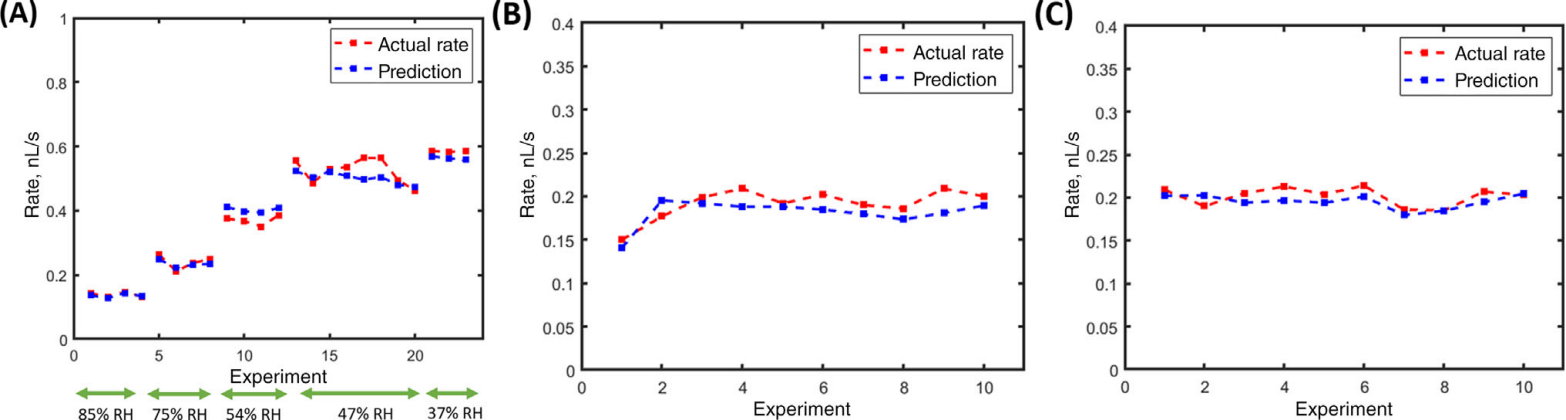
Comparison between the experiments and the prediction at various relative humidities (RH) on a glass slide for the model system (blue) and (A) water (red) (B) water + surfactant (red). (C) water + surfactant + high fructose corn syrup (red).

To calculate *D* in Eqn (6) for the conditions used in the study, 23 experiments were conducted on a glass slide with DI water under five different relative humidities (solution 1, Table 1) at 20 °C ($c_{v}$ = 0.0171 kg/m^3^, and ρ = 998.2 kg/m^3^). Using the least squares method, the best agreement between the experimental measurements of $\frac{\mathrm{dV}}{\mathrm{dt}}$ and the model prediction from Eqn (6) was achieved for a diffusion coefficient D = 1.95x10^−5^ m^2^/s, which is near the estimate of the vapor diffusion coefficient from Armstrong *et al*.^35^ The results are presented in Fig. 3(A). Similar experiments were conducted at 80% RH for solution 2 (water + surfactant) and solution 4 (water + surfactant + high fructose corn syrup), which showed good agreement between the model prediction using this calculated diffusion coefficient, as illustrated in Fig. 3(B),(C).

## 3 RESULTS AND DISCUSSION

### 3.1 Experimental results

**Figure 4:**
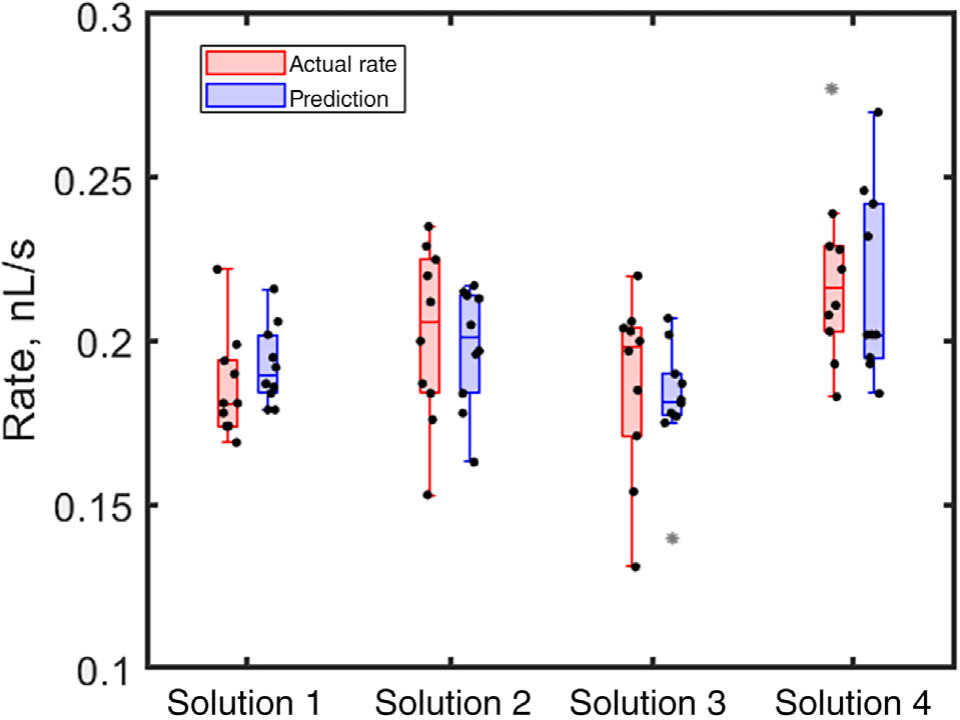
Whisker plots of volume loss rate for all the samples on cowpea leaves. For exact compositions see Table 1. Red bars show the actual rate while blue bars show the predicted rate. No statistical difference between the measurements for a given solution, indicating no uptake from cowpea leaves.

As the evaporation rates of the solutions are well characterized by the model predictions in the case of pure evaporation on a non‐porous substrate, these predictions can now be compared with the measurements of the volumetric loss rate of droplets on leaf surfaces conducted inside the relative humidity chamber. If the predicted rate is not statistically different from the actual rate, no uptake from the leaves is assumed; conversely, if the actual rate exceeds the predicted rate, a potential uptake can be considered. This study explores the potential novelty of using optical imaging methods, traditionally used for contact angle and evaporation rate measurements, to investigate and resolve uptake from leaves. A parameter called the degree of rate enhancement (DoRE) is defined, describing the difference between the predicted value for the evaporation of a sessile droplet and the measured rate of volume loss:

(7) $$\text{DoRE}=\left(\frac{\text{actual rate}}{\text{predicted rate}}-1\right)\cdot 100\%,$$

where the predicted rate is calculated using Eqn (6) for the initial contact angle. Initially, water droplets were tested on the surface of cowpea leaves (waxy). The presence of surfactant lowered the contact angle, shifting from 66° for water to 52.6° for water + surfactant, 49.4° for water + surfactant + LMO, and 49.5° for water + surfactant + high fructose corn syrup (Table 2). The results indicate that the volume change is evaporation‐dominated and there is negligible uptake by the leaf (Fig. 4), as the DoRE for all solutions is equal to or less than 3%, which is within the measurement error (a statistical metric based on variance in measurements) and can thus be neglected. Figure 4 was created using the boxchart function in MATLAB (The MathWorks Inc, Natick, MA, USA), where data points deviating more than 1.5 times the interquartile range from either the bottom or the top of the boxplot are considered outliers (shown as grey asterisks). These outliers are included in Table 2 for analysis.

**Table 2:** Experiments on cowpea leaf for different samples.

Solution	Solution composition	Initial contact angle, °	Actual rate of droplet volume loss on the leaf, nL/s	Predicted rate of droplet volume loss on the leaf, nL/s	Degree of rate enhancement, %
1	Water	66 (± 6.6)	0.193 (± 0.01)	0.186 (± 0.01)	−3.0 ( ±3)
2	Water + surfactant	52.6 (± 3)	0.198 (± 0.01)	0.202 (± 0.02)	1.7 ( ±4)
3	Water + surfactant	49.4 (± 4.3)	0.182 (± 0.01)	0.187 (± 0.02)	2.6 ( ±6)
4	Water + surfactant + high fructose corn syrup	49.5 (± 4)	0.217 (± 0.02)	0.22 (± 0.02)	1.6 (± 4.3)

For exact compositions see Table 1. Values in brackets are 95% confidence intervals based on variance in measurements.

**Figure 5:**
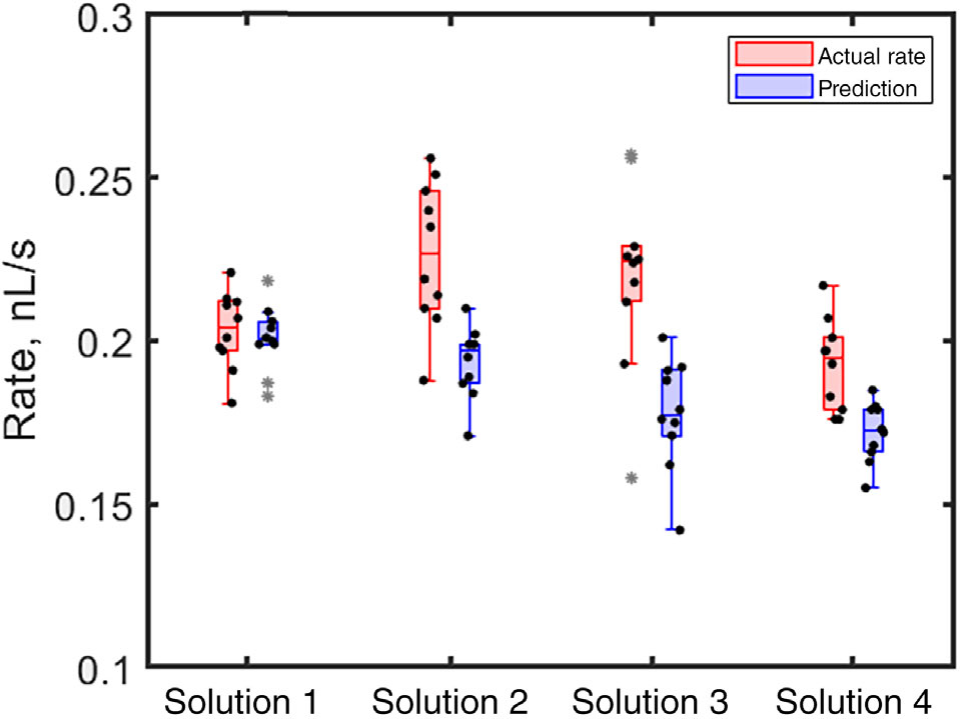
Whisker plots of volume loss rate for all the samples on sorghum leaves. For exact compositions see Table 1.

Subsequent experiments were conducted on the surface of sorghum leaves (hairy) to determine if they show similar behavior to cowpea leaves. Similar to cowpea leaves, no change in the rate was observed for water alone (Table 3 and Fig. 5). Additionally, like cowpea leaves, the presence of surfactant significantly reduced the initial contact angle, from 72.5° for water to 50.5° for water + surfactant, 54.5° for water + surfactant + LMO, and 55.9° for water + surfactant + high fructose corn syrup.

**Table 3:** Experiments on sorghum leaf for different samples

Solution	Solution composition	Initial contact angle, °	Actual rate of droplet volume loss on the leaf, nL/s	Predicted rate of droplet volume loss on the leaf, nL/s	Degree of rate enhancement, %
1	Water	72.5 (± 9.2)	0.203 (± 0.01)	0.203 (± 0.01)	1.4 (± 2.9)
2	Water + surfactant	50.5 (± 4.8)	0.227 (± 0.01)	0.194 (± 0.01)	16.9 (± 4.7)
3	Water + surfactant	54.5 (± 5)	0.22 (± 0.02)	0.178 (± 0.01)	23.7 (± 6.3)
4	Water + surfactant + high fructose corn syrup	55.9 (± 6)	0.193 (± 0.01)	0.172 (± 0.01)	11.9 (± 3.1)

For exact compositions see Table 1. Values in brackets are 95% confidence intervals based on variance in measurements.

However, the most surprising observation was not the change in contact angle but rather the consistent increase in rate on sorghum leaves compared to predictions based on evaporation alone (Table 3 and Fig. 5, see also Supporting Information, Fig. S2 for results with plants grown under different conditions). This result suggests possible uptake from the leaf. However, greater predicted rates may also occur because of the droplet spreading along the veins of a sorghum leaf, which can enlarge the wetted area and speed up the evaporation process.

### 3.2 Effect of spreading

**Figure 6:**
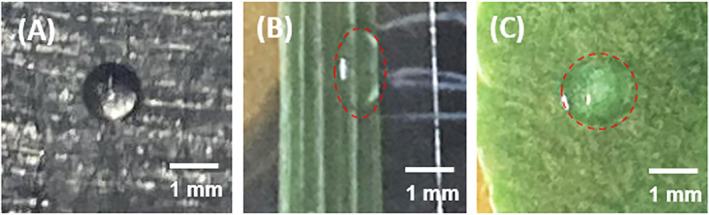
(A) No spreading on a glass slide. (B) Spreading sorghum (hairy) leaf along the veins. (C) Uniform spreading on cowpea (waxy) leaf.

**Figure 7:**
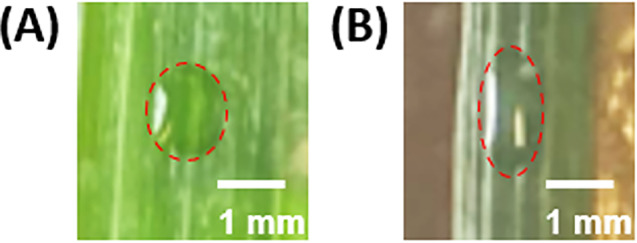
Example of lower and higher degrees of spreading on sorghum leaves. (A) Low spreading, contact area increase is 22%, (B) high spreading, contact area increase is 93%.

Unlike on a glass slide, where the droplet contact line typically remains stationary after deposition, on a leaf surface, droplets tend to spread. This spreading could potentially increase the uptake by the plant and the evaporation rate by changing the surface area of the droplet. When examining sorghum leaves, significant lateral spreading along the leaf veins was observed (Fig. 6(B)), while in cowpea leaves, more uniform spreading occurred (Fig. 6(C)). The increase in droplet contact area on the leaf surface was measured by capturing images using a phone camera (Fig. 7). It is important to note that the mathematical model (Eqn (6)) employed in the analysis for the evaporation rate does not consider spreading, which may result in the overestimation of the rate of volume loss. To determine if this is the case, the role of spreading on DoRE was evaluated.

Starting first with cowpea leaves, droplets of solution 1 (water) did not spread. For solution 2 (water + surfactant), the mean proportional increase in the diameter of the contact area on cowpea leaves was 8.7%, corresponding to an 18.2% increase in contact area. Similar results were obtained for solutions 3 and 4 (Table 4). Overall, the spreading results revealed up to a 20% change in the contact area, a relatively low or modest amount of spreading, especially compared to that on sorghum leaves, while no significant change to DoRE was observed (Table 2). This suggests that the model used to predict the evaporation rate is robust to a certain amount of spreading.

**Table 4:** Mean proportional increase in area coverage, given in %, for all samples on sorghum and cowpea leaves.

Solution	Solution composition	Mean area increase on cowpea, in %	Mean area increase on sorghum, in %
1	Water	0	0
2	Water + surfactant	20.5 (± 7.6)	65.1 (± 15.9)
3	Water + surfactant + LMO	16.9 (± 5.3)	40.3 (± 9.5)
4	Water + surfactant + high fructose corn syrup	20.5 (± 11.8)	59.7 (± 11)

For exact compositions see Table 1. Values in brackets are 95% confidence intervals based on variance in measurements.

Similar to cowpea, droplets of water (solution 1) did not spread on the surface of sorghum leaves. In contrast, droplets with the surfactant (solutions 2, 3, and 4) significantly increased the wetted area of sorghum in comparison with cowpea leaves. On average, the increase in the contact area is three times larger on sorghum leaves than on cowpea leaves (Table 4), although the specific increase depends on the solution type. The mean proportional increase of contact area for solution 2 (water + surfactant) was 65.1% (Table 4). Solution 4 showed a similar increase, with no statistical difference from solution 2. Correspondingly, both solutions showed the same increase in DoRE within the measurement uncertainty (Table 3). While this may implicate spreading as the primary cause of the changing DoRE, solution 3 breaks from this pattern by having the smallest change in contact area (although not significantly different from solutions 2 and 4) (Table 4) and yet having the largest increase in DoRE, statistically different from solution 4 (Table 3). This suggests that while some of the increase in DoRE may be attributed to spreading, the increase observed when LMO is present may be linked to actual uptake by the plant.

**Figure 8:**
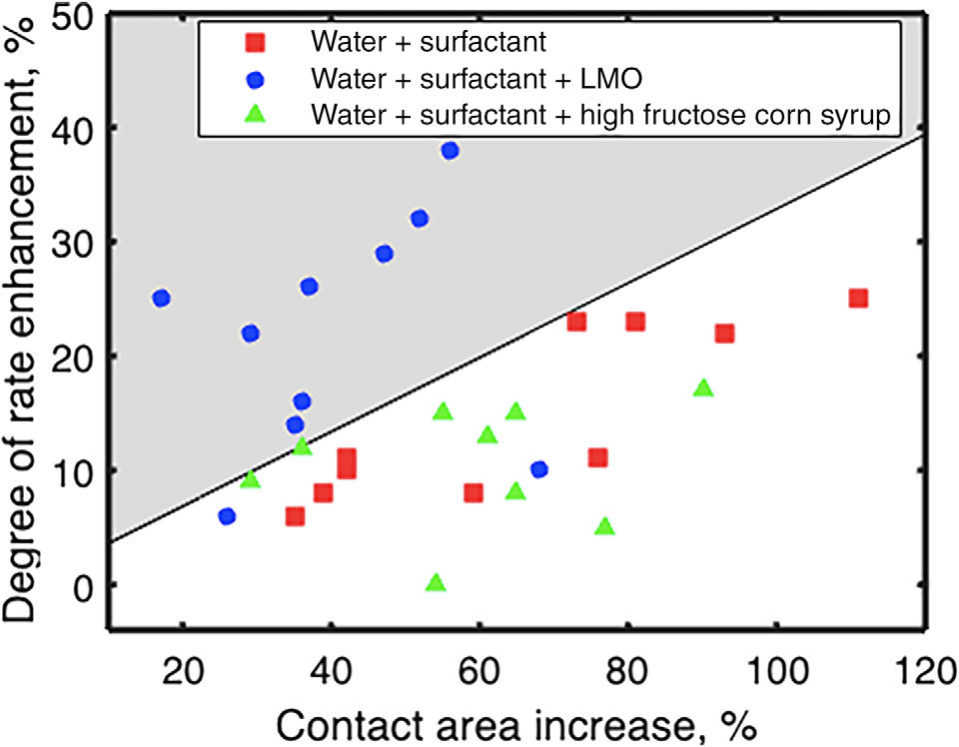
DoRE *vs* % contact area increase for all the samples on sorghum leaves. The grey area indicates samples with a higher rate than predicted for the equivalent spherical droplet, calculated from a simple model for the lateral spreading (Eqn (8)) and an assumed change in contact angle after spreading. Water + surfactant, solution 2; water + surfactant + LMO, solution 3; water + surfactant + high fructose corn syrup, solution 4.

To estimate whether the more substantial amount of spreading on sorghum affects the predicted rate from evaporation, the simple model system was applied to the current evaporation model to account for spreading and changes in shape. The final contact area of the droplet on the leaf after spreading was assumed to be an inner rectangle representing the ‘added’ area after spreading of width $2R_{0}$ and length $\left(L-2R_{0}\right)$, along with two semicircular caps on the sides with a radius equal to the initial radius of the droplet:

(8) $$S=\pi R_{0}^{2}+2R_{0}\left(L-2R_{0}\right)$$

where $R_{0}$ is the radius of the droplet after the deposition and L is the final length of the droplet after the spreading. $\pi R_{0}^{2}$ represents the initial area of the droplet after the deposition, $2R_{0}\left(L-2R_{0}\right)$ is the added area due to the spreading. The process of spreading typically occurs within 5–10% of the droplet's lifetime. For simplicity, it was assumed that the volume remains constant during spreading. After measuring the final area, the effective radius of the equivalent spherical droplet was calculated. Note that contact angle also changes if the droplet spreads while maintaining the same volume. To account for this change, a new height of the spherical cap (h) and a new contact angle (θ) were estimated, assuming a constant volume (0.2 μL) and the average $R_{0}$ for all samples with surfactant (613 μm). The new height was calculated using Eqn (3) and then substituted into Eqn (4) to determine the new contact angle. This contact angle was used to predict the evaporation rate using Eqn (6) and the new DoRE is shown as the line in Fig. 8. Results falling above this line in the grey section of the plot correspond to a higher volume loss rate than was predicted for the evaporation model for a spherical droplet with the same contact area based on the simple extended model. Most of the results involving water + surfactant + LMO (solution 3) are found in this section, indicating potential acceleration by leaf uptake. This is consistent with the literature, as mineral oils are known to increase absorption by a leaf.^39^, ^40^ An adjusted prediction was also made for droplets on cowpea leaves based on the final contact area, showing a negligible change to the initial prediction, and explaining the model's robustness to small/isotropic changes in spreading.

For each solution type, the increase in DoRE on sorghum generally tends to occur with the rise in the % increase of contact area. This correlation makes sense, as greater spreading results in a larger surface area covered by the same volume, resulting in a faster evaporation and potential uptake by a leaf. It should be noted that for sorghum plants grown under different conditions, different rates were observed (Supporting Information, Figs S2 and S3), but the trend holds that in general, the addition of surfactant alone (solution 2) increases spreading, and higher DoRE values are observed with more spreading. Conversely, the addition of light mineral oil in addition to surfactant (solution 3) does not spread as effectively but has significantly faster rates of volume loss, indicating potential uptake, while the addition of corn syrup and surfactant (solution 4) has modest spreading and rates of volume loss that correspond to that of evaporation alone.

## 4 CONCLUSIONS

In this work, a relative humidity‐controlled stage with accompanying imaging set‐up was used to test the evaporation and possible uptake of samples with surfactant, humectant, and accelerator on the surface of glass slides, sorghum (hairy), and cowpea (waxy) leaves. Samples incorporating surfactant, LMO, and high fructose corn syrup reduced the contact angle of droplets on waxy and hairy leaves, as expected. Pure water droplets did not spread on either type of leaf, whereas samples with surfactants resulted in a final wetted area more than 1.5 times as large as the initial one. For sorghum leaves, the DoRE parameter increased with the increasing % contact area, indicating an increase in the rate of volume loss with the larger surface area covered. However, samples with LMO did not spread as effectively compared to other surfactant‐laden solutions but increased the DoRE, suggesting an increase in volume loss may be due to uptake from the leaf. For waxy leaves, a minor spreading was observed with the presence of a surfactant, but it did not result in a change in DoRE. The findings suggest a complex interplay between adjuvants, droplet behavior, and plant physiology, warranting further investigation to clarify underlying processes.

Overall, the simplicity of the experimental system demonstrated herein enables a relatively straightforward assessment of the dynamics involved in the evaporation and uptake of a droplet on a leaf surface. Subsequent research using the system highlighted here could involve the addition of more agriculturally relevant samples, such as herbicides and foliar nutrients, and testing on plants grown under varied conditions, such as drought stress. Ultimately, this technology can aid in understanding the droplet dynamics and possible uptake on various plant surfaces under various environmental conditions particularly by researchers who may not have access to more invasive or complex methods, offering a simple and effective approach for studying these processes.

## Supporting information


**Data S1.** Supporting Information.

## Data Availability

The data that support the findings of this study are available from the corresponding author upon reasonable request.
