# Supplementary material for: Evaporation, spreading, and possible uptake of droplets on sorghum (Sorghum bicolor) and cowpea (Vigna unguiculata) leaves using an imaging‐based technology
Source: Pest Manag Sci. 2024 Oct 29;81(2):884–91. doi: 10.1002/ps.8491 (PMC11716365; doi:10.1002/ps.8491)
Supplement: Supplementary file 1 — Data S1. Supporting Information. [file PS-81-884-s001.pdf]

Supplementary material for

Evaporation, spreading and possible uptake of droplets on sorghum (*Sorghum bicolor)* and cowpea (*Vigna unguiculata)* leaves using an imaging-based technology

Iaroslav Makhnenko[1], Cody Hoerning[2], Dustyn D. Sawall[2], Steven A. Fredericks[2], Elizabeth R. Alonzi[2], Cari S. Dutcher[1,3]

[1]Department of Mechanical Engineering, University of Minnesota Twin Cities, Minneapolis, MN, USA
[2]Winfield United, River Falls, WI, USA
[3]Department of Chemical Engineering and Materials Science, University of Minnesota Twin Cities, Minneapolis, MN, USA

List of Figures

Figure S1: Sorghum plants under different growth conditions……………………………………S2

Figure S2: Whisker plots of rate for all the samples on sorghum leaves...……………………….....S3

Figure S3: DoRE vs.% contact area increase for all the samples on sorghum leaves.…………..S4

Figure S4: Predicted vs. actual rate for attached and detached leaves……………...…………..S5

1. Potential Impact of growth conditions on sorghum plant experiments

Additional experiments were conducted on sorghum leaves under different growth conditions (**Fig. S1**) using solutions #2, #3, and #4 (see **Table 1**). The new set of plants spent only 8 days in a greenhouse before being transported to a laboratory for testing, in contrast to the previous plants that spent 21-25 days in a greenhouse. The results from the new plants showed higher DoRE for all the solutions (green circles in **Fig. S2**) with the same increase in contact area (**Fig. S3**). These findings suggest that growth conditions and age of plants may impact the DoRE and potentially affect uptake from sorghum leaves.

Note that sorghum plants in **Fig. S1A** appear to be at the approximately five-leaf stage, while the plants in **Fig. S1B** appear to be at two-leaf stage. Sorghum is known to undergo significant leaf morphological changes at the five/six-leaf stage with the change in density of trichomes (1). The changes observed in **Fig. S2** and **Fig. S3** may be in-part due to the sorghum phase transition.

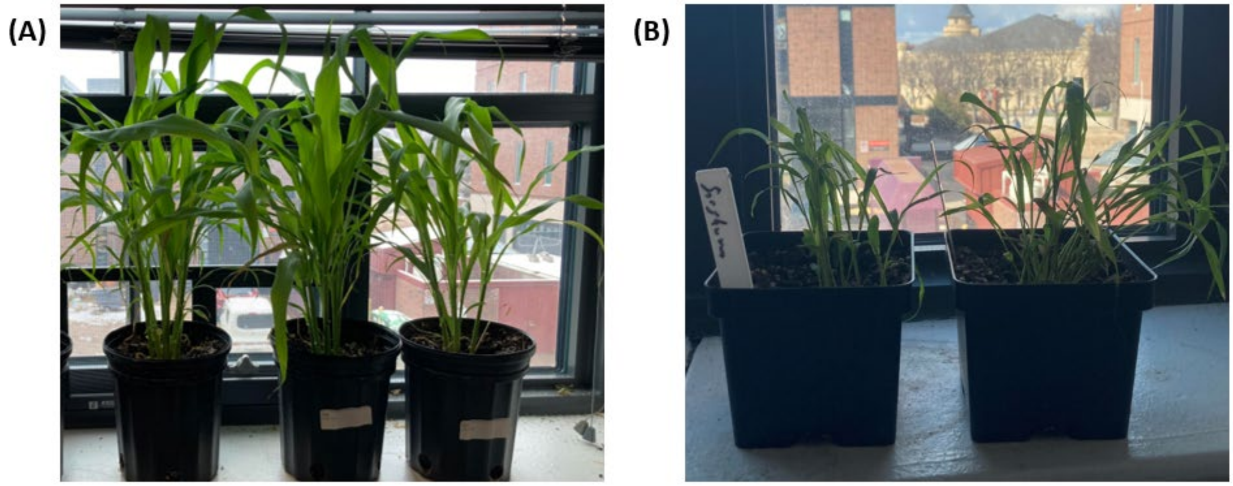

Figure S1. (A) Sorghum plants grown for 21-25 days in a greenhouse. (B) Sorghum plants grown for 8 days in a greenhouse

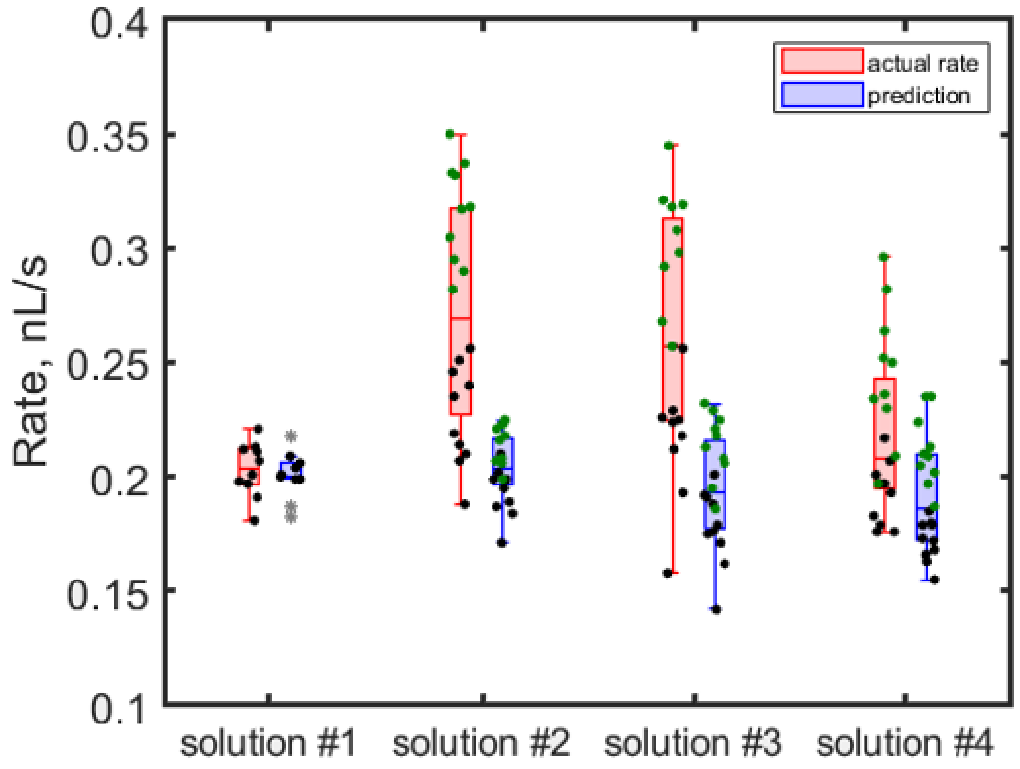

Figure S2. Whisker plots of rate for all the samples on sorghum leaves. Grey asterisks show the outliers. Black circles correspond to experiments conducted on leaves that spent 21-25 days in a greenhouse (Figure S1A), while green circles represent experiments on leaves that spent 8 days in a greenhouse (Figure S1B).

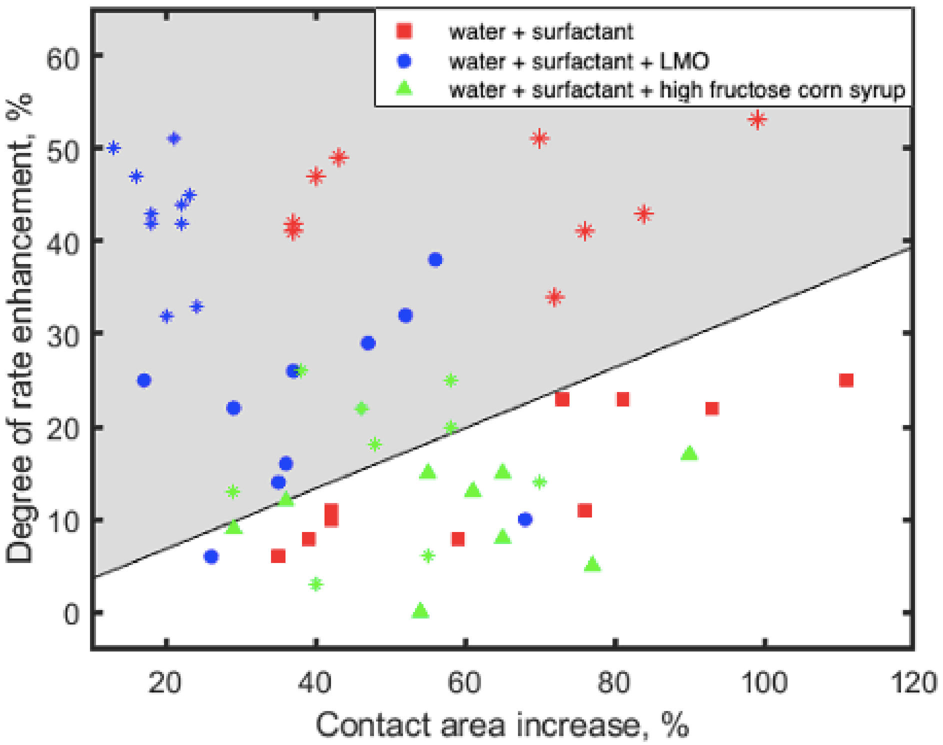

Figure S3. DoRE vs.% contact area increase for all the samples on sorghum leaves. The grey area indicates samples with a higher rate than predicted for the equivalent spherical droplet, calculated from a simple model for the lateral spreading (Eq. 8) and an assumed change in contact angle after spreading. Squares, circles, and triangles correspond to experiments conducted on leaves that spent 21-25 days in a greenhouse (Figure S1A), while asterisks represent experiments on leaves that spent 8 days in a greenhouse (Figure S1B).

2. Attached vs. detached leaves

Experiments at ~80% RH (same as the original experiments) were conducted with solution #2 (water + surfactant) on the surface of sorghum leaves to investigate whether detached leaves yield similar results as attached leaves (**Fig. S4**). A t-test conducted at $\alpha = 0.01$ for both attached and detached leaves does not reject the null hypothesis with p-value less than 0.06 for both predicted and actual rates, indicating that there is no significant difference between experiments on attached and detached leaves.

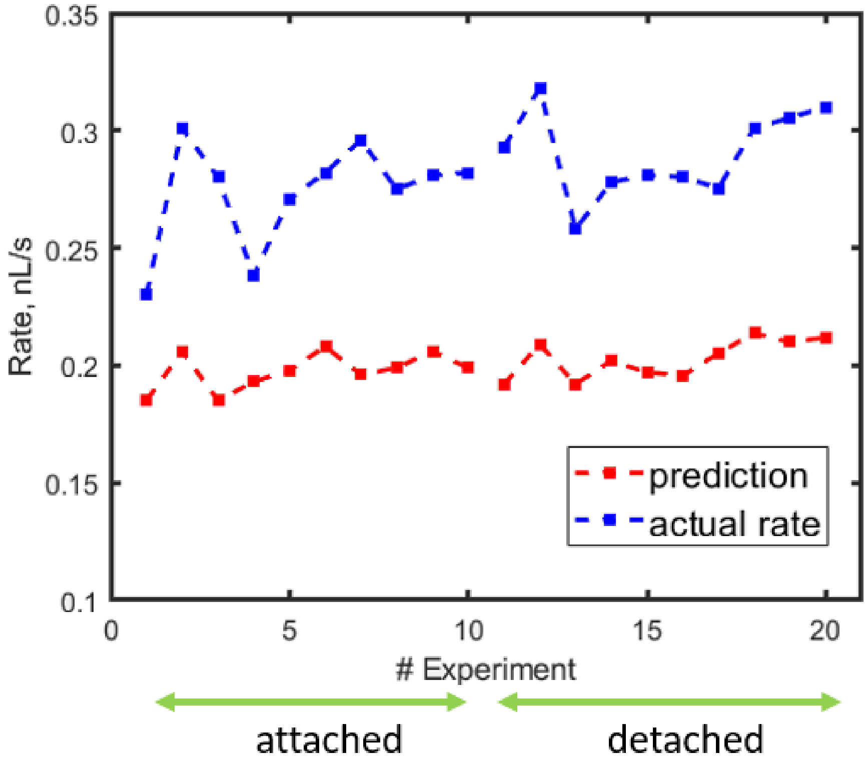

Figure S4. Comparison between the experiments and the prediction for attached and detached leaves. No significant differences between the sets according to the t-test.

References

1. Hashimoto S, Tezuka T, Yokoi S. Morphological changes during juvenile-to-adult phase transition in sorghum. Planta. 2019 Nov 1;250(5):1557–66.
